# In Vitro Aberrometric Assessment of a Multifocal Intraocular Lens and Two Extended Depth of Focus IOLs

**DOI:** 10.1155/2017/7095734

**Published:** 2017-11-29

**Authors:** Vicente J. Camps, Angel Tolosa, David P. Piñero, Dolores de Fez, María T. Caballero, Juan J. Miret

**Affiliations:** Department of Optics, Grupo de Óptica y Percepción Visual (GOPV), Pharmacology and Anatomy, University of Alicante, Alicante, Spain

## Abstract

**Purpose:**

To analyze the “in vitro” aberrometric pattern of a refractive IOL and two extended depth of focus IOLs.

**Methods:**

A special optical bench with a Shack-Hartmann wavefront sensor (SH) was designed for the measurement. Three presbyopia correction IOLs were analyzed: Mini WELL (MW), TECNIS Symfony ZXR00 (SYM), and Lentis Mplus X LS-313 MF30 (MP). Three different pupil sizes were used for the comparison: 3, 4, and 4.7 mm.

**Results:**

MW generated negative primary and positive secondary spherical aberrations (SA) for the apertures of 3 mm (−0.13 and +0.12 *μ*m), 4 mm (−0.12 and +0.08 *μ*m), and 4.7 mm (−0.11 and +0.08 *μ*m), while the SYM only generated negative primary SA for 4 and 4.7 mm apertures (−0.12 *μ*m and −0.20 *μ*m, resp.). The MP induced coma and trefoil for all pupils and showed significant HOAs for apertures of 4 and 4.7 mm.

**Conclusions:**

In an optical bench, the MW induces negative primary and positive secondary SA for all pupils. The SYM aberrations seem to be pupil dependent; it does not produce negative primary SA for 3 mm but increases for higher pupils. Meanwhile, the HOAs for the MW and SYM were not significant. The MP showed in all cases the highest HOAs.

## 1. Introduction

The correction of presbyopia is a very popular issue in the world. The increasing of working life and the visual stress, determined by the electronic devices, have also made patients more demanding. The introduction of MF IOLs (multifocal intraocular lenses) as a refractive tool in ocular surgery has been determined by the aim to provide a good vision from near to intermediate distances (considering the reading of papers or electronic devices) making presbyopic patients spectacle independent. According to this purpose, bifocal IOLs improved far and near or intermediate vision on the basis of their optical addition, while trifocal IOLs represented a step forward by the increasing of the number of the optical foci [1]. Unfortunately, the concept of multifocality is the cause of the occurrence of photic phenomena related to light scattering and of the increasing of high order aberrations (HOAs). Patient dissatisfaction was expressed in spite of the achievement of a visual rehabilitation in terms of distance, intermediate, and near visual acuities [2]. EDOF IOLs (extended depth of focus IOLs) may be considered as a new generation of IOLs. They have been engineered in order to provide a continuous vision, simulating the natural lens, thus covering the vision from near to far without significant loss of quality of vision and also reducing the onset of visual disturbances. One solution to this problem is based on the control of HOAs (high order aberrations). HOAs may influence the quality of vision, and also they have been recently shown that they may increase the depth of focus of the eye and therefore provide a good functional intermediate and near vision [3, 4]. Moreover, the wavefront analysis has been widely used to detect the effects of lower and higher order aberrations and their contribution to the optical quality in both in vitro and in vivo sets [5].

The aim of this study is to analyze the “in vitro” aberrometric pattern of a new extended depth of focus IOL, Mini WELL (SIFI, Italy), comparing it with that obtained with the extended range of vision diffractive IOL TECNIS Symfony ZXR00 (Abbott Laboratories, Illinois, USA) and with the asymmetric rotationally refractive multifocal IOL Lentis Mplus X LS-313 MF30 (Oculentis GmbH, Berlin, Germany). The Mini WELL is a new progressive IOL, in which the primary and secondary spherical aberrations that are negligible at the pupil center of a real eye in normal conditions are induced in an appropriate amount in some specific areas of the IOL optics, providing an increase of the depth of focus and a control of HOAs. Some in vitro studies have shown to this date that this model of IOL is able to provide good levels of optical quality compared to different multifocal IOLs [6, 7]. In particular, it has been demonstrated that Mini WELL has a better optical quality than the TECNIS Symfony IOL at far vision and a larger defocus tolerance than the diffractive lens at near vision. Mini WELL also assures a better optical performance compared to trifocal IOLs, and in clinical trial, it has revealed a continuous and progressive vision from near to far with a negligible occurrence of visual disturbances [8].

## 2. Methods

### 2.1. Description of IOLs Measured

**Figure 1:**
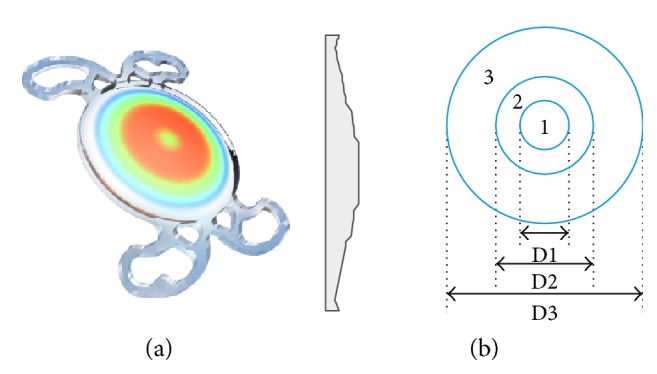
(a) Picture MINI WELL IOL. (b) The optical design: the inner (D1) and middle (D2) zones have different spherical aberrations with opposite signs; the outer one (D3) is an aspherical zone.

The Mini WELL (Figure 1(a)) is considered as a progressive extended depth of focus intraocular lens (EDOF IOL) with an equivalent addition of +3.0 D. The optical design is based on application of positive and negative spherical aberrations in the central part of the lens, in order to increase the depth of focus and to generate a “continuum range of focus.” The optic is divided into three different annular zones: the inner and middle zones have different spherical aberrations with opposite signs, whereas the outer one is a monofocal aspherical zone (see Figure 1(b)). The lens' overall diameter is 10.75 mm, its optical surface diameter is 6 mm, and it includes an ultraviolet filter. The dioptric spectrum is from 0 to +30 D. In our in vitro study, we used an IOL with 20 D of optical power.

The manufacturer describes the TECNIS Symfony ZXR00 as a biconvex and pupil-independent diffractive IOL, which combines an achromatic diffractive surface with an echelette design. Its overall diameter is 13.0 mm, and its optical zone diameter is 6.0 mm. The power spectrum available ranges from +5.0 to +34.0 D and incorporates an ultraviolet (UV) light-absorbing filter. The power of the TECNIS Symfony ZXR00 IOL we used was 20 D, with an addition of +4.00 D. The Lentis Mplus X LS-313 MF30 IOL is described as a refractive varifocal IOL composed by an aspheric distance vision zone combined with a 3.00 D posterior sector-shaped near-vision zone allowing <seamless varifocal transition between the zones. Its overall diameter is 11.0 mm, and its optical zone diameter is 6.0 mm. The power spectrum available ranges from −10 to +36.0 D. In our study, we used an IOL with a power of 20 D and an addition of 3 D.

### 2.2. Measurement Experimental Setup

The WFS150-5C Shack-Hartmann wavefront sensor (Thorlabs, Germany) was used for the measurement of the aberratic profile of the three IOLs. This wavefront sensor is available with a chrome-masked microlens array for use in the 300–1100 nm range with a lenslet pitch of 150 *μ*m and a maximum aperture size of 5.95 × 4.76 mm.

**Figure 2:**
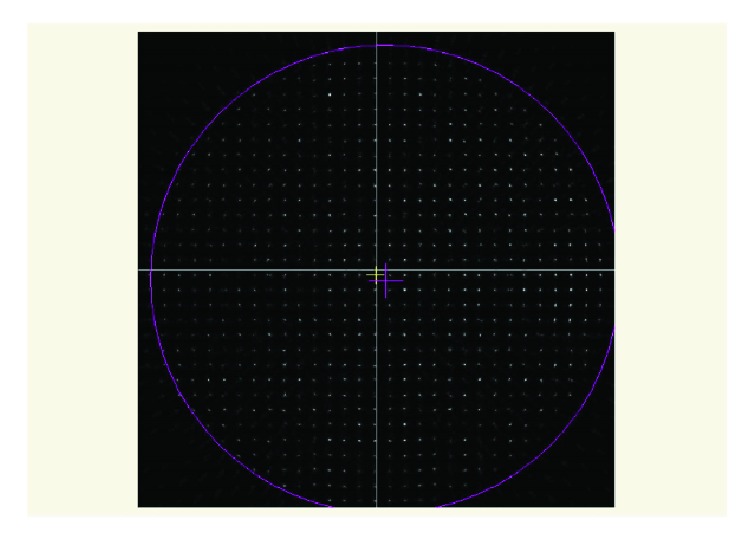
Spotfield of the Symfony for a pupil size of 4.7 mm.

In spite of some authors question the validity of the Shack-Hartmann in the measurement of diffractive IOLs [8–11], these drawbacks are minimized with the TECNIS Symfony ZXR00 (see Figure 2). The diffractive zones of the TECNIS Symfony ZXR00 are large enough (only 10 diffractive zones) to be resolved by our configuration (lenslet pitch of 150 *μ*m and a low wavelength 532 nm [11]). There are some isolated spots not well defined due to the microlens that are registering the wavefront from a diffractive transition zone. In addition, the other two of the IOLs had an optical refractive design.

**Figure 3:**
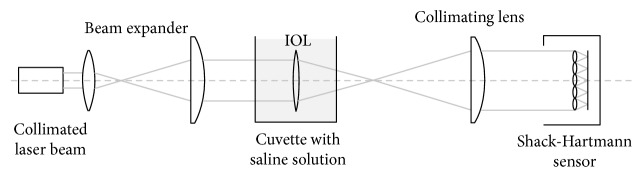
Optical layout.

Figure 3 shows the optical layout used for measuring the wavefront aberrations of the multifocal IOLs. The system consists of a diode-collimated laser beam of 532 nm, a beam expander, a wet cell in which the IOL was submerged, a collimating lens, and a Shack-Hartmann wave-front sensor. The wet cell is a chamber that has transparent optical windows on its top and bottom and is filled with lens solution (0.9% normal saline). The IOL is placed on the bottom optical window of the wet cell. An *XYZ* translational stage is attached to the wet cell to align the IOL with the optical axis of the wavefront sensor.

We have measured the aberrometric pattern in the exit pupil plane of the three IOLs. Only Zernike polynomials from the third to sixth orders were considered. Three measurements of the aberrometric pattern were done for each IOL, and the mean value was obtained for each Zernike coefficient. The temperature of the cuvette with saline solution was 35° for all the measurements, and three different pupil sizes were used for the comparison: 3, 4, and 4.7 mm. The maximum pupil size we can measure is determined by the Shack-Hartmann sensor which is in this case 4.76 mm.

## 3. Results

**Figure 4:**
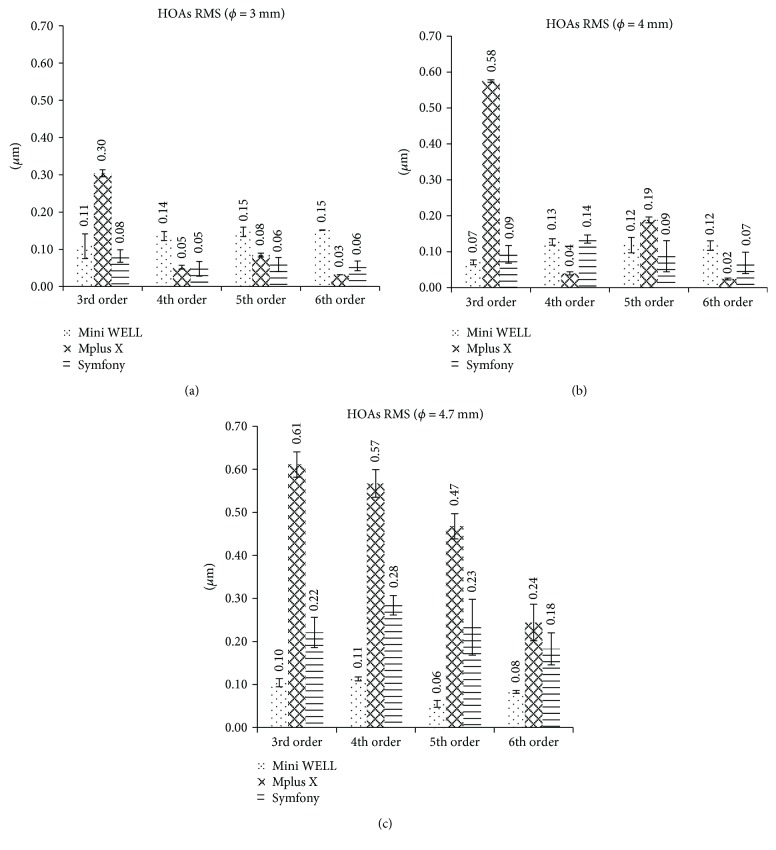
Root mean square with standard deviation (RMS ± SD) by Zernike orders for the three IOLs as a function of the pupil (MW: Mini WELL ready; MP: Lentis Mplus X LS-313 MF30; SYM: TECNIS Symfony ZXR00): (a) for 3 mm, (b) for 4 mm, and (c) for 4.7 mm.

Table 1 shows all the Zernike coefficients obtained for each IOL and for each pupil aperture, and Figure 4 displays the root mean square (RMS) values associated to high order aberrations (HOAs) and to the different Zernike orders.

When an aperture of 3 mm was considered, the Mini WELL IOL showed the highest 4th, 5th, and 6th RMS orders, due to the negative primary spherical aberration of −0.13 ± 0.01 *μ*m and positive secondary spherical aberration of +0.12 ± 0.02 *μ*m. Likewise, the highest 5th RMS order value was observed due to the secondary horizontal coma of 0.121 ± 0.001 *μ*m. The Lentis Mplus X LS-313 MF30 IOL showed a higher value of the 3rd order RMS (0.30 ± 0.01 *μ*m) compared to the Mini WELL due to the presence of coma and trefoil. The RMS values for the rest of orders were low. The TECNIS Symfony ZXR00 IOL showed low RMS values for all Zernike orders (<0.1 *μ*m).

For an aperture of 4 mm, the Lentis Mplus X LS-313 MF30 IOL showed the highest 3rd order RMS (0.58 ± 0.01 *μ*m) caused by the presence of coma (0.40 ± 0.01 *μ*m) and trefoil (0.41 ± 0.01 *μ*m). This IOL showed a 5th order RMS of 0.19 ± 0.01 *μ*m. The Mini WELL-ready IOL showed similar values for the 4th and 6th order RMS values (0.12 *μ*m) due to the relatively similar magnitude of negative primary spherical aberration (−0.12 ± 0.01 *μ*m) and positive secondary spherical aberration (+0.08 ± 0.02 *μ*m) induced by the IOL. The TECNIS Symfony ZXR00 IOL only showed a remarkable RMS value for the 4th order (0.14 *μ*m) due to the presence of negative primary spherical aberration (−0.120 ± 0.006 *μ*m).

Finally, when a pupil aperture of 4.7 mm was considered, the Lentis Mplus X LS-313 MF30 IOL showed very high values of the 3rd, 4th, and 5th order RMS values (0.61 ± 0.03 *μ*m, 0.57 ± 0.03 *μ*m, and 0.47 ± 0.03 *μ*m, resp.). For the 6th order, the RMS value was also important (0.24 ± 0.04 *μ*m). The Mini WELL-ready IOL showed the lowest values for all RMS orders. The TECNIS Symfony ZXR00 IOL showed values between 0.18 ± 0.04 *μ*m for the 6th order RMS and 0.28 ± 0.02 *μ*m for the 4th order RMS. For the Symfony IOL at this pupil size, also the contribution of trefoil, primary spherical aberration, and tetrafoil were important (+0.20 ± 0.05 *μ*m, −0.20 ± 0.02 *μ*m, and +0.15 ± 0.02 *μ*m, resp.).

Considering the overall HOAs, RMS (see last row of Table 1) for a pupil aperture of 3 mm, the Lentis Mplus X LS-313 MF30 IOL showed the highest values (0.32 ± 0.02 *μ*m), while the TECNIS Symfony ZXR00 IOL showed the lowest value (0.11 ± 0.06 *μ*m). The Mini WELL IOL showed a RMS value of 0.25 ± 0.06 *μ*m. For a pupil aperture of 4 mm, the Lentis Mplus X LS-313 MF30 IOL showed again the highest HOA RMS value (0.61 ± 0.02 *μ*m). The Mini WELL and the TECNIS Symfony ZXR00 IOLs showed similar HOA RMS values (0.21 ± 0.05 *μ*m and 0.16 ± 0.06 *μ*m, resp.). For the pupil aperture of 4.7 mm, the Lentis Mplus X LS-313 MF30 IOL showed again the highest HOA RMS value for high order aberrations (1.0 ± 0.1 *μ*m) while the Mini WELL IOL showed the lowest (0.18 ± 0.02 *μ*m). For this same pupil size, the TECNIS Symfony ZXR00 IOL showed a RMS value very close to 0.5 *μ*m (0.4 ± 0.2 *μ*m).

## 4. Discussion

The optical quality and behavior of a MF IOL may be studied through objective and subjective methods which belong to preclinical and clinical experimental sets. Only a few studies have evaluated in vitro the aberrometric behavior of multifocal IOLs. In this paper, we have proposed a simple method of measurement based on a Shack-Hartmann sensor and considering only the IOL in wet conditions without an artificial cornea (see Figure 3). With this method, we are able to characterize the aberrometric profile of each IOL and to predict prior to surgery the potential impact of this profile on the visual performance of eyes implanted with them. Furthermore, the method has been valid for the three IOLs studied, including the diffractive IOL, because as we have previously justified our setup produced an acceptable spot field for the TECNIS Symfony ZXR00 IOL (see Figure 2). Other in vitro methods used for IOL characterization only provides an assessment of the visual quality, but without a complete description of the aberrometric profile.

Our results showed that for a 3 mm pupil, the Mini WELL IOL is the only IOL that, as expected according to the optical design, generates negative primary and positive secondary spherical aberrations (−0.13 *μ*m and +0.12 *μ*m, resp.). In fact, this result is consistent with the optic design of the lens provided by the manufacturer (see Figure 1). Specifically, the IOL provided a combination of positive and negative spherical aberrations to obtain an increased depth of focus. Benard et al. demonstrated by means of adaptive optics that the combination of primary and secondary spherical aberrations of opposite sign could increase the depth of focus more than three times for pupils larger than 4.5 mm [12]. This pattern of combination of negative primary and secondary positive spherical aberrations (−0.12 *μ*m and +0.08 *μ*m) was also observed with the Mini WELL IOL for pupil aperture of 4 mm. For this pupil aperture, the extended range of vision of the TECNIS Symfony ZXR00 IOL generated some negative primary spherical aberration (−0.12 *μ*m) to compensate the positive primary spherical aberration which is normally present in the cornea. Concerning the Lentis Mplus X LS-313 MF30 IOL, it was shown to induce vertical coma, trefoil, and pentafoil. It should be considered that this specific type of refractive IOL has another basis to increase the depth of focus, which is the induction of some levels of coma. This type of aberration has been shown to be able to increase the depth of focus significantly [4].

For 4.7 mm pupil aperture, a similar trend was observed. Mini WELL IOL induced again negative primary spherical aberration and some positive secondary spherical aberration (−0.12 *μ*m and +0.08 *μ*m), and TECNIS Symfony ZXR00 IOL generated negative primary spherical aberration (−0.20 *μ*m). The Lentis Mplus X LS-313 MF30 IOL generated coma and trefoil, as well as some high level of other 4th, 5th, and 6th order aberrations. The TECNIS Symfony ZXR00 IOL induced somewhat level of 4th, 5th, and 6th order aberrations higher than MW and lower than MP IOLs.

This aberrometric behavior of the Mplus IOL is consistent with the clinical data reporting the result of the subtraction of corneal to total aberrations in vivo [13, 14].

To this date, there are no clinical studies reporting the in vivo aberrometric outcomes with the Mini WELL and Tecnis Symfony IOLs. However, the results obtained in our experience with the TECNIS Symfony ZXR00 IOL are consistent with the in vitro evaluations of the aberrations obtained in the optical bench by Gatinel and Loicq [15]. In this study that was performed with the NIMO TR0815 instrument (Lambda-X), these authors found a primary spherical aberration of −0.05 *μ*m for 3 mm pupil size and −0.2388 *μ*m for 4.5 mm pupil size. The NIMO TR0815 device uses an artificial cornea in air for the measurement, and this can be the reason we obtained lower values of spherical aberration. Furthermore, our study confirmed the pupil dependency of the aberrations generated by the TECNIS Symfony ZXR00 IOLs since we obtained the aberrations increased when the pupil increased in size.

We have not found any study evaluating in vitro the aberrations of the Lentis Mplus X LS-313 MF30 IOLs, but different clinical studies with the Lentis Mplus LS-312 IOL reports, as previously commented, significant levels of intraocular aberrations. Specifically, significant levels of intraocular horizontal and vertical coma and spherical aberration have been reported in a great variety of clinical studies [5, 13, 14, 16]. Our results are consistent with these previous studies. Furthermore, we have also found quadrafoil, pentafoil, secondary trefoil, and secondary coma in the Lentis Mplus.

Finally, it can be concluded that for the Lentis Mplus X LS-313 MF30 and TECNIS Symfony ZXR00 IOLs the aberrations increased as the pupil size increased since +0.98 *μ*m and 0.42 *μ*m of overall HOAs, respectively. On the contrary, the level of HOAs was maintained within a physiological range [17] (between 0.18 and 0.25 *μ*m of overall HOAs) with an increasing pupil size with the Mini WELL IOL. The inner and middle zones are generating different spherical aberrations with opposite signs, whereas the outer one is a monofocal aspherical zone and does not induce aberrations; consequently, it is expected that the aberrations are already present for small optical diameters and are maintained as the optical diameter is larger. Our results corroborate this assumption.

A possible limitation of our study is the measurement of the aberration pattern of diffractive multifocal IOLs with a Shack-Hartmann wave sensor; therefore, the use of our optical bench design for this type of IOLs will require further studies. Anyway, as it was commented above, these possible drawbacks were not found for the TECNIS Symfony ZXR00. An artificial cornea has not been considered in the optical bench, but the results can be used for simulating the effect of the aberrations generated by the IOLs in any theoretical eye, analyzing the possible effects. In addition, it is important to mention that these results were obtained “in vitro” and they would be confirmed in future clinical studies.

In the next study, we would like to predict “in vitro” the effect of the aberrations generated by multifocal lenses in some eyes with aberrated corneas, trying to find out if they provide an optimum level of intraocular optical quality and are a good option for these eyes.

## Figures and Tables

**Table 1:** Zernike coefficients and standard deviation (±SD) in microns obtained for the three IOLs and for each exit pupil size (MW: Mini WELL; MP: Mplus; SYM: TECNIS Symfony ZXR00). The last row shows the HOAs.

Zernike coefficients (*μ*m)										
		*ϕ*=3 mm			*ϕ*=4 mm			*ϕ*=4.7 mm		
Aberration name	C (*n*, *m*)	MW	MP	SYM	MW	MP	SYM	MW	MP	SYM
*Vert trefoil*	C (3, −3)	−0.05 ± 0.07	0.200 ± 006	−0.04 ± 0.02	0.02 ± 0.02	0.41 ± 0.01	−0.03 ± 0.04	0.06 ± 0.02	0.41 ± 0.02	0.06 ± 0.03
*Vert coma*	C (3, −1)	0.05 ± 0.01	0.22 ± 0.02	−0.05 ± 0.01	0.05 ± 0.02	0.40 ± 0.01	0.05 ± 0.01	0.083 ± 0.001	0.27 ± 0.02	0.03 ± 0.04
*Horiz coma*	C (3, 1)	−0.030 ± 0.006	−0.010 ± 0.006	0.037 ± 0.005	0.01 ± 0.01	−0.007 ± 0.001	0.00 ± 0.08	0.01 ± 0.01	−0.31 ± 0.03	−0.04 ± 0.02
*Horiz trefoil*	C (3, 3)	0.040 ± 0.001	0.045 ± 0.003	−0.01 ± 0.04	0.04 ± 0.02	0.080 ± 0.007	0.00 ± 0.03	0.011 ± 0.003	0.17 ± 0.06	0.20 ± 0.05
*Vert tetrafoil*	C (4, −4)	−0.004 ± 0.004	−0.044 ± 0.007	−0.01 ± 0.03	0.00 ± 0.01	−0.01 ± 0.02	0.01 ± 0.02	0.038 ± 0.004	0.20 ± 0.03	0.04 ± 0.03
*Sec vert astigmatism*	C (4, −2)	0.006 ± 0.001	0.011 ± 0.006	0.02 ± 0.01	0.002 ± 0.001	−0.02 ± 0.01	0.01 ± 0.02	0.003 ± 0.003	−0.37 ± 0.02	0.03 ± 0.04
*Spherical aberration*	C (4, 0)	−0.13 ± 0.01	0.018 ± 0.008	−0.006 ± 0.004	−0.12 ± 0.01	0.022 ± 0.009	−0.120 ± 0.006	−0.105 ± 0.005	−0.29 ± 0.02	−0.20 ± 0.02
*Sec horiz astigmatism*	C (4, 2)	0.02 ± 0.02	0.008 ± 0.001	−0.002 ± 0.009	0.03 ± 0.01	−0.007 ± 0.003	0.01 ± 0.03	0.006 ± 0.003	−0.09 ± 0.04	0.11 ± 0.05
*Horiz tetrafoil*	C (4, 4)	0.01 ± 0.02	−0.01 ± 0.01	0.04 ± 0.02	0.01 ± 0.02	−0.002 ± 0.006	0.055 ± 0.005	−0.013 ± 0.005	0.22 ± 0.03	0.15 ± 0.02
*Vert pentafoil*	C (5, −5)	0.04 ± 0.03	0.073 ± 0.008	0.02 ± 0.04	0.01 ± 0.02	0.178 ± 0.008	0.01 ± 0.05	0.012 ± 0.008	0.21 ± 0.05	0.07 ± 0.07
*Sec vert trefoil*	C (5, −3)	−0.02 ± 0.04	−0.019 ± 0.006	−0.03 ± 0.01	−0.02 ± 0.02	−0.024 ± 0.008	−0.04 ± 0.03	0.01 ± 0.01	−0.23 ± 0.02	0.00 ± 0.01
*Sec vert coma*	C (5, −1)	−0.01 ± 0.05	−0.021 ± 0.007	0.017 ± 0.005	−0.009 ± 0.006	−0.045 ± 0.005	0.04 ± 0.05	0.013 ± 0.004	−0.23 ± 0.01	0.1 ± 0.1
*Horiz sec coma*	C (5, 1)	0.121 ± 0.001	−0.007 ± 0.005	−0.025 ± 0.007	0.078 ± 0.008	−0.005 ± 0.005	0.017 ± 0.007	0.049 ± 0.008	−0.252 ± 0.006	0.09 ± 0.03
*Horiz sec trefoil*	C (5, 3)	0.029 ± 0.007	0.001 ± 0.005	0.00 ± 0.01	0.07 ± 0.02	−0.012 ± 0.004	0.01 ± 0.01	−0.004 ± 0.005	0.01 ± 0.01	0.07 ± 0.08
*Horiz pentafoil*	C (5, 5)	0.0059 ± 0.006	0.03 ± 0.01	0.00 ± 0.02	0.05 ± 0.03	0.031 ± 0.008	−0.02 ± 0.04	0.00 ± 0.01	0.04 ± 0.02	0.1 ± 0.1
*Vert hexafoil*	C (6, −6)	0.01 ± 0.03	−0.024 ± 0.004	−0.03 ± 0.03	−0.01 ± 0.02	−0.019 ± 0.005	0.00 ± 0.02	0.01 ± 0.01	0.036 ± 0.007	0.07 ± 0.09
*Sec vert tetrafoil*	C (6, −4)	0.016 ± 0.007	0.005 ± 0.003	0.01 ± 0.02	0.01 ± 0.02	−0.003 ± 0.005	0.00 ± 0.04	−0.007 ± 0.005	−0.091 ± 0.006	−0.01 ± 0.04
*Tert vert astigmatism*	C (6, −2)	0.009 ± 0.002	−0.008 ± 0.002	0.02 ± 0.009	−0.026 ± 0.009	0.011 ± 0.002	0.026 ± 0.008	0.014 ± 0.008	−0.11 ± 0.01	0.06 ± 0.08
*Sec spherical aberration*	C (6, 0)	0.12 ± 0.02	0.013 ± 0.004	0.01 ± 0.01	0.08 ± 0.02	−0.001 ± 0.005	0.02 ± 0.05	0.079 ± 0.003	−0.14 ± 0.02	0.03 ± 0.03
*Tert horiz astigmatism*	C (6, 2)	0.06 ± 0.03	0.006 ± 0.003	0.01 ± 0.02	0.038 ± 0.006	0.002 ± 0.001	−0.03 ± 0.02	0.008 ± 0.003	0.00 ± 0.03	0.04 ± 0.05
*Horiz sec tetrafoil*	C (6, 4)	0.01 ± 0.05	0.009 ± 0.002	0.018 ± 0.009	0.05 ± 0.02	0.005 ± 0.002	0.00 ± 0.02	0.008 ± 0.001	−0.06 ± 0.03	0.07 ± 0.02
*Horiz hexafoil*	C (6, 6)	0.03 ± 0.04	−0.004 ± 0.005	0.01 ± 0.02	0.01 ± 0.05	0.001 ± 0.006	−0.008 ± 0.005	−0.003 ± 0.004	0.11 ± 0.05	−0.02 ± 0.08

Overall HOAs	RMS	0.25 ± 0.06	0.32 ± 0.02	0.11 ± 0.06	0.21 ± 0.05	0.61 ± 0.02	0.16 ± 0.06	0.18 ± 0.02	1.0 ± 0.1	0.4 ± 0.2
